# The Experiences of Women with Gestational Breast Cancer: Perspectives of Healthcare Professionals

**DOI:** 10.1177/01939459261475787

**Published:** 2026-09-07

**Authors:** Sara Hurren, Marie Mcauliffe, Karen Yates, Tracey Ahern, Cate Nagle

**Affiliations:** 1 College of Healthcare Sciences, James Cook University, Cairns, QLD, Australia; 2 College of Healthcare Sciences, James Cook University, Townsville, QLD, Australia; 3 Centre for Quality and Safety Research, Barwon Health Partnership, Deakin University, Geelong, VIC, Australia

**Keywords:** breast cancer, women’s health, pregnancy, nursing, healthcare professionals, qualitative research

## Abstract

**Background:**

Gestational breast cancer (GBC) is defined as breast cancer diagnosed during pregnancy or in the first 12 months post-partum. The diagnosis and management of GBC in the prenatal and postnatal settings pose significant challenges for women, their families, and healthcare professionals (HCPs).

**Objective:**

The objectives of this study were to explore women’s experiences of GBC from the perspectives of HCPs.

**Methods:**

This qualitative exploratory study involved 11 HCPs in Australia, including 7 nurses, a midwife, an oncologist, a breast surgeon, and a psychology counsellor. Data were gathered through semi-structured individual interviews, which were transcribed verbatim and analysed thematically using Braun and Clarke’s method. Participants were recruited from November 2021 to June 2022.

**Results:**

The data analysis identified four themes: (1) “It’s always very traumatic for them,” (2) “It’s quite a different experience compared to other women with breast cancer,” (3) “They are often people who need quite a bit of support,” and (4) “These women do have to make different treatment decisions.”

**Conclusion:**

This exploratory study offers valuable insights from HCPs who have cared for women with GBC, shedding light on their unique journeys. The findings highlight the distinct challenges that set women with GBC apart from other breast cancer patients, emphasising specific psychosocial support needs. Moreover, the study reveals that the decision-making process for these women is powered by essential elements such as knowledge, support, and trust.

## Introduction

While the prevalence of gestational breast cancer (GBC) is relatively rare, over recent decades, there has been an increase in the incidence of women from high-income countries being diagnosed with GBC, due, in part, to maternal age increasing as women delay childbearing.^1-4^ The definition of GBC is a breast cancer diagnosed during pregnancy or within 12 months post-partum.^1,2,5^ The diagnosis of GBC accounts for 6%–15% of breast cancer cases in women aged 20–44 years.^2^ Despite GBC being rare, it is one of the most common types of malignancies occurring during pregnancy and lactation (about 1 in 3000 pregnancies).^1,6^

A diagnosis of GBC displays unique molecular and epidemiological traits.^2^ GBC tumours are typically more aggressive, with a notable family history of breast and ovarian cancers and germline mutations in BRAC1/2.^1,2^ GBC cases are more prevalent in younger women, with about 50% of cases classified as triple-negative breast cancers.^1^ GBC is a distinct entity with specific clinical and molecular features that may require improvements in risk assessment, diagnosis, and treatment.^1,2^

GBC requires specific medical, psychological, and sociological support from healthcare systems.^7,8^ Some of the clinical challenges involve diagnosing and staging GBC. Frequent delays in diagnosis are due to differences between breast changes during pregnancy and those that may indicate a tumour, highlighting the need for specialised multidisciplinary teams for effective treatment.^9^

Prenatal care for women with GBC is complex due to risks associated with imaging and fetal safety, impacting decisions about the timing and mode of delivery.^9^ Post-partum, breastfeeding is generally contraindicated during treatment.^9^ Other treatment difficulties experienced by healthcare professionals (HCPs) relate to the clinical presentation of GBC, which includes high-risk features such as young age at diagnosis, large tumour size, high tumour grade, extensive lymph node involvement, and a higher risk of specific tumour subtypes.^7^ Treating GBC requires coordinated efforts from a range of multidisciplinary clinicians and healthcare teams.^10^

The psychological management of GBC and multidisciplinary collaboration pose challenges for caring for pregnant patients.^10^ Centralising care and following up-to-date, evidence-based guidelines are difficult due to the rarity of GBC and the limited expertise.^9^ While there are national strategies in Australia aimed at improving maternity and oncological care, guidelines for managing cancer during pregnancy are lacking.^10^ Harris et al^11^ and Stafford et al^10^ have highlighted gaps in the provision of psychological care for women with gestational cancers. Key issues include the need for tailored interventions and training for HCPs, improved multidisciplinary collaboration, access to expert HCPs, and designated clinical liaison roles to facilitate communication between obstetrics and oncology services.^10^ In addition, access to peer support and resources is crucial for informing women with GBC.^10,11^

Each woman with GBC is unique, requiring individualised planning in oncology, surgery, radiation, and obstetrics to assess the risks of treatments for both the mother and the baby, as not all women will need every type of treatment. Women diagnosed with GBC often face heightened psychological distress when considering available treatment and management options.^12^ The psychological challenges faced by women with GBC include how HCPs communicate with them, which can significantly impact their handling of the GBC diagnosis and the decision-making process involving treatment options.^13-15^

Effectively addressing the complexities of a GBC diagnosis and treatment may involve utilising specialised centres in both public and private hospitals to ensure that all necessary services are available for seamless care and management.^16^ While our understanding of the psychological needs of women with GBC is gradually improving, there remains limited published literature about HCPs’ views and knowledge of the experiences of women with GBC.

## Purpose

There are a limited number of studies describing the perspectives of HCPs who have cared for women with GBC. This research aimed to deepen the understanding of the challenges these women face from a clinical viewpoint. Specifically, it sought to illuminate the interactions between women with GBC and contemporary healthcare systems. The insights gained aimed to enhance clinical practice, research, and education, ultimately improving support and communication for these women and their families. Following completion of a scoping review,^17^ the authors developed an exploratory qualitative study specifically to address the research question: What are the experiences of women with GBC from the perspectives of HCPs? The findings help identify psychological support needs and improve communication strategies for future care delivery.

## Methods

### Study Design

This qualitative, exploratory study examined the experiences of women with GBC from HCPs’ perspectives. The researcher used a qualitative methodology within an interpretivist framework to explore and analyse the experiences of women with GBC from HCPs’ perspectives, enabling the researcher to identify potential areas for improvement to better support and strengthen women with GBC within the healthcare system. The study is reported in accordance with the Consolidated Criteria for Reporting Qualitative Research, a 32-item checklist designed explicitly for interviews and focus groups.^18^

### Participants

A guiding principle in qualitative research is that sampling is a deliberate process of selecting participants who can provide rich data on the phenomenon of interest, and that sampling should continue only until data saturation has been achieved.^19^ In this study, the experiences of a specialist group of HCPs were explored. In the recruitment process, convenience and snowball sampling were used to identify eligible participants with recent clinical care experience for women diagnosed with GBC. Anyone meeting the inclusion criteria was eligible; no exclusion criteria were applied.

The study included healthcare clinicians registered with the Australian Health Practitioner Regulation Agency who had cared for women diagnosed with GBC in Australia within the past 10 years. Recruitment involved contacting national professional organisations and websites for medical and nursing groups, including obstetricians, gynaecologists, midwives, breast cancer nursing groups, psychologists, and general practitioners.

### Data Collection

Qualitative data were collected from November 2021 to June 2022. Due to COVID-19, interviews were conducted via phone and recorded. The researcher emailed a participant information sheet to HCPs, detailing the study’s purpose and consent process. Interested participants contacted the researcher for clarification before scheduling interviews, and verbal consent was obtained after the information was explained.

After obtaining consent, the researcher conducted private phone interviews using open-ended and semi-structured interview questions to gather information. Field notes were recorded immediately after each interview. An interview guide (Table 1) facilitated the process and addressed the research question. Participant interviews were recorded as MP3 files. A transcription service converted the audio files into Word documents, which the researcher verified for accuracy by comparing the transcripts with the recordings. The transcripts were then transferred to an Excel spreadsheet, with additional columns added for analysis.

**Table 1.** Interview Guide for Healthcare Professionals.

The first few questions I will ask the participant to provide demographic data and to break the ice. How many years have you been employed in your profession? What is the scope of your current role?
Questions
1. Tell me about your experience of caring for women with GBC?
2. How did the women navigate their way through the healthcare system?
3. What did you think worked well?
4. What do you think did not work well?
5. In your opinion, what aspects of care or systems could be improved for women with GBC?
6. Is there anything further you would like to add?

Abbreviation: GBC, gestational breast cancer.

### Data Analysis

A thematic analysis framework by Braun and Clarke,^20^ featuring six distinct phases, was used for data analysis. This structured approach ensured a thorough exploration of the data.^21^ Thematic analysis is a flexible method for identifying patterns and themes, enhancing understanding of the research findings.^20^

In Phase 1, the researcher reviewed all the interviews by listening to the recordings and reading and re-reading the transcripts. During this process, brief notes were taken on any relevant words, ideas, or insights for each interview and the overall dataset. In Phase 2, coding involved systematically assigning meaningful descriptions or labels to data segments. Any discrepancies in coding were discussed with the research advisory team (CN, MM, KY, and TA) and resolved by consensus, resulting in clearer code definitions to ensure reliability.

In Phase 3, all generated codes were identified to reveal shared patterns across the dataset and were carefully checked. Sub-themes and themes were developed from the participants’ ideas, feelings, and perceptions. In Phase 4, the development and review of themes included verifying that the themes made sense in relation to both the other coded extracts and the entire dataset. Finally, in Phase 5, the analysis continued with further refinement, definition, and naming of the themes. The last phase involved writing a report summarising the HCPs’ views on women’s experiences with GBC. Examples of the HCPs data analysis process, from meaning units to themes, are presented in Table 2.

**Table 2.** Examples of Healthcare Professionals’ Analysis Process from Meaning Units into Themes.

Meaning units	Codes	Sub-themes	Themes
So really challenging with emotions, where you should be so joyous and excited, and then you’ve got this fear, fear and anxiety that comes with that (H4C19).	Fear, anxiety, and emotional reaction	Heightened emotions	“It’s always very traumatic for them”
Women are thinking about their own mortality with a newborn (H8C48).	Fear of death	Fear of loss	
A woman in this situation with GBC, there may only be one woman that’s been in the same situation with them (H2C55).	Different to other women	Women feel isolated	“It’s quite a different experience compared to other women with breast cancer”
She found the lump on the day that she was told her pregnancy was—she was pregnant; she’s doing this on her own until she feels that she’s into that first trimester (H4C25).	Timing of diagnosis	She didn’t have time to stop and breathe	
	Lump found at the same time of pregnancy.		
	Doing this on her own		
… the management of the neonate, the separation from the neonate, the fact that they can’t breastfeed is a distress for the child and the mother, and then, caring for the neonate when the mother needs care herself (H11C76).	Separation from baby	Added layer of complexity	
	Unable to breastfeed		
	Distressing child and mothers’ needs		
They’re really often people that are going to need quite a bit of support. Not just for them but for their partner and their family because of the shock of the situation (H2C26).	Need for support		“These are often people who will need quite a bit of support”
	Women		
	Partner		
	Family		
One woman decided to have surgery and then wouldn’t have any more treatment. She decided to go an alternative route which was really heartbreaking. She wouldn’t have chemotherapy (H5C45).	Decisions about surgery		“These women do have to make different treatment decisions”
	Choices about treatment		
	Advocating for the baby and herself		
	Alternative route		

Abbreviation: GBC, gestational breast cancer.

Alphanumeric coding and line numbers were assigned to the HCPs, using the letter (H) followed by numbers 1 to 11, and a C with a corresponding cell line number within the spreadsheet (eg, H7C25). This de-identified coding facilitated systematic identification and organisation.

In this qualitative study, sampling concluded upon reaching data saturation, at which point no further data yielded new insights. The recruited participants provided expert insights into the challenges faced by women diagnosed with GBC. The researcher thoroughly read and re-read each transcript, identifying codes and examining them for new aspects or distinctions. The goal was to gather rich, detailed accounts and insights from key HCPs.

### Ethical Considerations

On October 19, 2020, the Human Research Ethics Committee at James Cook University, Australia, granted ethical approval for this study (approval: H8236). Data access was restricted to the research advisory team, and all identifiable participant information was removed from the reported findings.

## Results

Thirteen HCPs agreed to be interviewed; however, one did not meet the selection criteria, and another withdrew. A total of 11 participants with diverse clinical backgrounds and geographical locations participated in this study. Each interview lasted between 18 and 52 minutes, with a median of 29 minutes; no repeat interviews occurred. Table 3 presents the demographic characteristics of participants.

**Table 3.** Demographic Characteristics of HCPs.

HCPs	Geographical location	Occupation	Years of experience stated in 2020-2021
HCP1	Victoria Regional	BCN	Nurse Midwife since 1980
			20 years of experience
HCP2	Queensland Regional	Oncology Nurse	Nursing 39 years/doing Oncology Nursing 34 years
HCP3	Queensland Metropolitan	Metastatic BCN	Oncology nurse 20 years, BCN 10 years
HCP4	Victoria Metropolitan	BCN	BCN 12 years
HCP5	Western Australia Regional	BCN	BCN 8 years
HCP6	Adelaide Metropolitan	BCN, CNC	CNC role 2.5 years, 15 years of experience
HCP7	Victoria Metropolitan	Metastatic BCN	BCN 24 years
HCP8	Western Australia Metropolitan	Psychological counsellor	Counselling support for 6 years, breast cancer support for 4 years
HCP9	Queensland Regional	Breast surgeon	Consultant surgeon 15 years of experience
HCP10	Queensland Regional	Midwife	Midwife with 30 years of experience
HCP11	Tasmania Regional	Medical oncologist	Generalised medical oncologist 16 years

Abbreviations: BCN, breast care nurse; CNC, clinical nurse consultant; HCP, healthcare professionals.

Through analysis of the data provided by the HCPs participating in the study, four main themes emerged (Figure 1). The themes are as follows: (1) “It’s always very traumatic for them,” (2) “It’s quite a different experience compared to other women with breast cancer,” (3) “They are often people who need quite a bit of support,” and (4) “These women do have to make different treatment decisions.”

**Figure 1.**
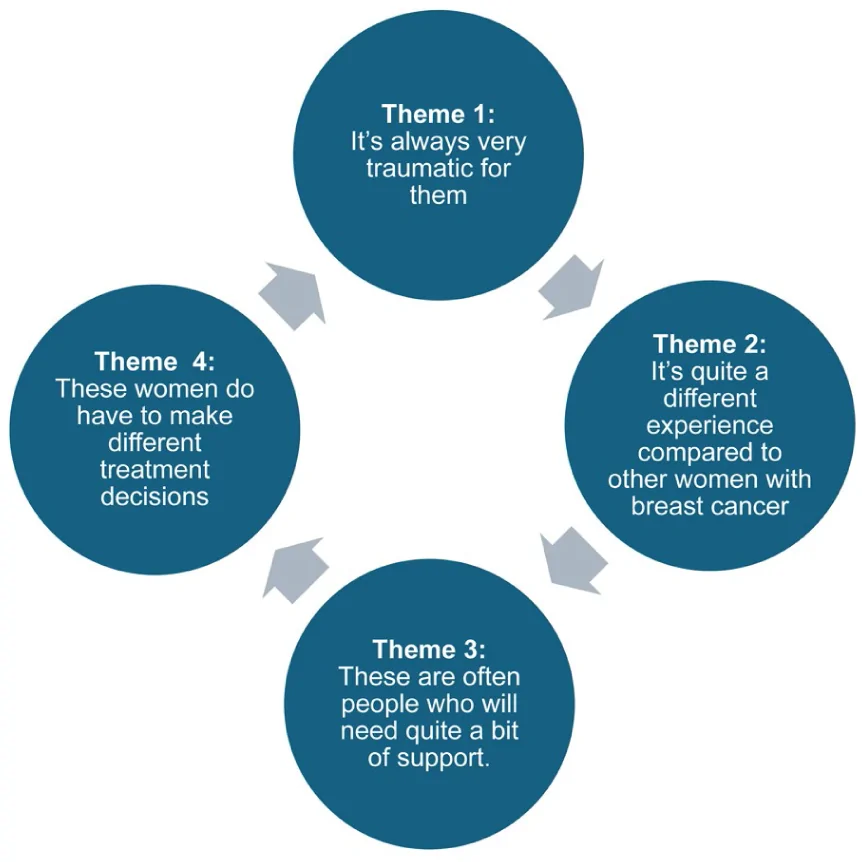
Themes: women’s experiences of gestational breast cancer: perspectives of healthcare professionals.

### Theme 1: “It’s Always Very Traumatic for Them”

The narratives from HCPs provided insights into the experiences of women diagnosed with GBC and the direct impact on their lives. They reported that the women encountered significant psychological trauma and challenges in adjusting to their diagnosis. The impact on the women began upon receiving their diagnosis and continued throughout their treatment and afterward. The HCPs said that it was particularly stressful during different stages of pregnancy and treatment. The HCPs described some of the women as mothers in crisis.

She is dealing with a cancer diagnosis—a terminal cancer diagnosis. It was horrific. (H3C39)

The HCPs stated that the psychological effects on the women were notably difficult. Many experienced heightened emotions, anxiety, distress, and fear. Some women faced particularly intense challenges of shock and horror when diagnosed shortly after undergoing in vitro fertilisation, soon after giving birth, or when confronted with terminal cancer. The HCPs noted the reaction of disbelief of these women, especially in the first trimester or so soon after giving birth, and many of the women were unaware that breast cancer could occur during pregnancy.

It was heightened emotions, heightened emotions the whole way through. (H4C42)

HCPs have observed that trauma can create intense fear and a sense of loss for women facing GBC. This includes concerns about cancer recurrence, the possibility of losing their pregnancy, the threat of metastatic disease, and fears about death. Many women experience heightened anxiety during uncertain times, while others worry that their treatments might negatively affect their baby’s health. Additionally, HCPs have reported that many women express concerns about not being able to be present for their children and families.

That instant feeling of, I might—or the fear of, will I be around to see this baby grow up? (H8C19)

### Theme 2: “It’s Quite a Different Experience Compared to Other Women with Breast Cancer”

HCPs have noted that women with GBC face unique challenges that are different from other breast cancer patients. This complexity stems from the rarity of GBCs, which means these women often lack opportunities to meet others in similar situations. Another factor setting them apart is their age; they are typically young women who are either pregnant or have recently given birth. This places them at a different stage in their lives compared to other breast cancer patients, who are often diagnosed later in life.

You don’t see a lot of women with gestational breast cancer obviously, compared to the number of other women with breast cancer. (H2C17)

HCPs have noted that women diagnosed with GBC are often in the early stages of family planning and may have specific concerns regarding fertility, breastfeeding, genetic testing, and difficult decisions about termination of pregnancy. As a result, these women sometimes feel emotionally disconnected and socially isolated because of the timing of their diagnosis and the stage of their lives.

HCPs have also highlighted that this sense of isolation also extends to their support systems, which are significantly different. Most existing breast cancer support groups typically cater to older demographics, which makes it challenging for younger women with GBC to find relatable support. As a result, HCPs have observed that these women frequently struggle to connect with support that is aligned to their unique situations.

Would not find anyone else in that group in a similar situation as themselves or would be very unlikely to. (H2C53)

Feeling very different to other mothers, feeling different to other women their age. (H8C41)

HCPs have observed that women facing GBC experience the most significant challenges during the early stages of pregnancy and in making treatment decisions. This critical period involves quick decision-making, and it has been reported that women often feel overwhelmed by the complexity and urgency of the communication from multiple clinical teams, as well as by the detailed information about their diagnosis, staging, pregnancy, cancer genetics, and treatment.

The urgency to commence treatment often varies with the stage of the cancer and the type of treatment used. In contrast to the typical experiences of breast cancer patients, the HCPs noted that the women with GBC require immediate attention. This urgency arises from the need to consider potential risks to the fetus, the diagnosis itself, fertility issues, motherhood, breastfeeding, and responsibilities that come with caring for a newborn. The HCPs recalled that this pressing decision-making process left women feeling as though they had no time to pause and reflect, making it difficult to prepare for their decisions.

… They need to be treated urgently .... (H2C17)

She had her baby and then was told you’ve only got three months if you don’t do anything. (H3C28)

Healthcare providers have observed that women’s experiences are extremely complex. These complexities can include managing the physical demands of treatment while caring for a newborn, facing difficulties with breastfeeding, dealing with childcare issues during hospital stays, navigating relationship challenges, and making treatment decisions. For example, women often deliberate over their options for surgery, such as whether to pursue prophylactic mastectomies or to undergo bilateral mastectomies followed by reconstruction, which adds to the complexity of their decisions. Additionally, women must consider factors such as scheduling treatment, timing of surgeries, types of surgeries, timing of chemotherapy before childbirth, and the timing of delivery.

She had a unilateral mastectomy so she could have breastfed from the other side, but breastfeeding is difficult at the best of times, so I’m sure that it added a layer of complication for her. (H6C42)

### Theme 3: “They Are Often People Who Need Quite a Bit of Support”

HCPs have identified that women diagnosed with GBC require additional support during their diagnosis. They have noted both strengths and weaknesses within the healthcare system concerning this support. The strengths include the involvement of midwives, breast care nurses, psychologists, social workers, oncologists, surgeons, and other essential individuals, including women’s partners and families. However, a significant challenge these women faced was navigating both the healthcare system and available community resources. This situation forces them to independently coordinate services and develop support networks to effectively address their ongoing needs.

.... It all ends on the poor woman, trying to keep tabs on everything. (H9C21)

It was identified by the HCPs that when immediate family members cannot provide support, women often seek help from friends, social media, and practical resources like breast milk donation and childcare services.

She just gathered all her friends around her, and they used social media to help get a group together and support her. (H5C69)

HCPs observed that the partners of women diagnosed with GBC can either offer support or may themselves need psychological assistance.

It’s obviously a lot of psychological stress for the partner. (H11C58).

While another HCP observed that women try to protect their partners by shielding them from further emotional turmoil.

I think she’s doing that, she says that she’s doing that for him, so that he’s emotionally not—found the losses, the pregnancy loss is incredibly hard, so for her it’s not wanting to tell him something that potentially will hopefully cause great joy, but then all of a sudden there could be a loss, another loss .... (H4C77)

### Theme 4: “These Women Do Have to Make Different Treatment Decisions”

The HCPs expressed that some women are actively engaged in the care, treatment, and management of GBC, while others are not. During this critical period, some women were able to clearly express their preferences and needs. These decisions included selecting the location for childbirth, choosing between private and public healthcare facilities, or opting for a combination of both, deciding when to start treatment as their birth approaches, considering alternative treatments, and determining which healthcare providers would be involved in their care.

They get kind of, not forced, but they have to kind of make a difficult decision, I guess. (H6C44)

The HCPs observed that many women sought information from various sources to support their decision-making. These decisions are influenced by the woman’s level of trust and comfort with the healthcare system, even when the facility does not provide maternity services. The quote below discusses the interplay between personal values and the information available to individuals. It highlights a specific perspective on lactation, suggesting that the option to maintain it was not adequately presented.

She could have maintained lactation too. I don’t think that was really offered. (H1C34)

## Discussion

This study explored the experiences of women with GBC from the perspectives of 11 HCPs across various clinical backgrounds and geographic locations in Australia. The experiences identified by the HCPs were grouped into four themes. HCPs have consistently noted that the journey faced by women with GBC, from diagnosis to treatment, can be extremely challenging from a psychological perspective. This trauma arises not only from the physical difficulties associated with the disease but also from the emotional strain of managing two conditions simultaneously. Cancer can be profoundly traumatic for some patients, leading to psychological issues like post-traumatic stress disorder.^22^

Our findings, consistent with various qualitative and quantitative studies in Australia and internationally, indicate that HCPs are aware that women with GBC often experience a state of emotional crisis. These women are in a hypersensitive state, enduring extreme distress and are overwhelmed.^10,12,14,23^ Despite the use of various measures of psychological distress in these studies, the results consistently indicate that a cancer diagnosis causes significant emotional distress for pregnant women.^23^ Kyriakides^24^ and other researchers support these, emphasising the importance of examining the broader effects of these psychosocial experiences, both in the short and long term.^12,24^

The experiences of women with GBC bring forth feelings of loss related to motherhood, fear of death, and emotions that become more intensified during pregnancy.^25^ A study by Ives et al^26^ aligns with our findings and highlights that women facing GBC have distinct perspectives on motherhood and their desire for more children, which, in turn, affects their chances of survival.^26^ The fear of not witnessing their children’s growth is a significant source of distress that influences their treatment decisions.^26^ A study by Faccio et al^25^ supports our findings and states that due to this loss of control over their lives, these women may struggle to find the mental space to consider their maternity, as their focus is primarily on their illness and its possible effects on their child.^25^ While it is evident that a cancer diagnosis during this critical time can lead to extreme distress, it also raises concerns about whether there are enough psychosocial supports available to help them.^10^ This emotional trauma aspect must be fully considered in the care and support provided to these women.

The findings demonstrated that HCPs perceived the journey for these women to be markedly different. Several factors contribute to this unique set of challenges and experiences, including the aggressive nature of the cancer, the urgency of treatment decisions, and specific medical complications. However, for the women, the experience is different due to the rarity of the disease, with limited peer support structures and a disconnection from other women with GBC. These findings align with other qualitative studies in that women often perceive themselves as exceptional cases, mainly due to the rarity of gestational cancers.^16,26-28^ This perception is further complicated for HCPs in connecting the women with others who have undergone similar experiences, who are mothers or in similar life stages.

Findings reveal that HCPs have noted specific challenges faced by women, particularly related to age and responsibilities in early family planning and caring for newborns. Key concerns differ among women with GBC, especially in relation to fertility preservation, the effects of carrying a genetic mutation, treatment options, and interruptions to breastfeeding.

A systematic review by Leung et al^29^ found findings similar to this current study, identifying two themes, “lost opportunity” and “not fitting in.” These themes indicate women with gestational cancers are impacted by healthcare decisions that influence their delivery plans, concerns about future fertility, and the effects of lost breastfeeding opportunities.^29^ Although there is a lack of studies specifically addressing the different aspects of diagnosis and treatment for women with GBC, existing research suggests that available resources predominantly cater to older women. This discrepancy is particularly noticeable in areas such as fertility preservation, breastfeeding support, treatment-related menopause, and overall parenting needs.^16,26,29^

Our findings show that HCPs recognise that women with GBC face distinct physical changes and limitations compared to those with breast cancer. This is especially true for women unable to breastfeed after a mastectomy or those nursing from one breast post-unilateral mastectomy. Several qualitative studies support these observations, revealing that many women experience a heightened sense of difference or inadequacy in their roles as mothers, which is associated with more significant physical changes and with limited resources to meet these unique requirements, compared with those observed in healthy or pregnant women.^27,30,31^

The HCPs have observed that women with GBC experience unique challenges compared to other women with breast cancer, which are closely related to psychological distress and their support needs. The HCPs observed that these support systems are difficult to navigate and that the women often took a leading role in managing their support needs and accessing their social networks. Several studies align with this finding and emphasise that accessing appropriate psychosocial supports remains a significant barrier for women with GBC.^11,16,29,31^ Many existing cancer support groups are often perceived as unsuitable for younger women, who may feel disconnected and different because many cancers are more commonly diagnosed in older populations.^26,31^ This disconnect raises significant questions about the inclusivity and relevance of current support frameworks.

In Australia, women with GBC must navigate care that can be provided at different hospitals, across public or private settings, geographical locations, and diverse medical and surgical specialists.^10^ While much of the literature reports the need for more diverse and tailored peer support groups for women with GBC, it also points out that women with GBC experience not knowing about available services or resources.^11,16,29^ Several other studies support this by aligning with our findings in highlighting that centralising referrals, creating clinical liaison roles, and connecting women with peer support will alleviate the stress and anxiety.^10,31^

This study found that women with GBC face significant challenges in the decision-making process. The HCPs observed that women with GBC face different treatment decisions compared to other women. These decisions are highly personal, such as choosing a particular surgeon, and are influenced by family obligations or by what is best for the baby. This, in turn, contributes to their psychological distress and trauma.

Additionally, there is still a need for further education and training in the complexities of GBC and gestational cancers. While several studies support our findings that women rely on HCPs for emotional support, psychological services, expertise, and communication that assist their decision-making, this raises questions about whether current healthcare systems can adequately meet these needs in practice.^10,26,29,32^

The four identified themes were closely interconnected and collectively influenced by HCPs’ perceptions of women’s experiences with GBC. The HCPs have observed that the central element linking these themes for women diagnosed with GBC is that they often experience significant feelings of isolation and uncertainty. These healthcare providers reported that women with GBC frequently feel different from other women with breast cancer, which leads to unique psychological responses and challenges, particularly concerning motherhood. The HCPs noted that the decision-making process for GBC patients is complex, which can heighten anxiety and increase the need for support. These findings may provide HCPs with insights for more empathetic and personalised care, ultimately enhancing the well-being of women diagnosed with GBC.

### Limitations and Future Recommendations

A significant limitation of this study was the lack of face-to-face interviews, essential for establishing rapport and trust. In-person interactions enable the observation of nonverbal cues and the collection of more detailed information. Due to COVID-19, the study relied on phone interviews. While convenience sampling and snowball sampling are effective qualitative methods, they also have significant limitations.^33,34^ Sampling bias occurs when self-selected participants share similar characteristics, resulting in limited diversity. Additionally, perspective bias may arise from an unbalanced sample, resulting in overrepresentation of certain viewpoints and the underrepresentation of others, thereby limiting the breadth and diversity of perspectives captured. While the study offers valuable insights into women’s experiences with GBC, a broader range of HCP perspectives may have provided additional understandings.

HCPs involved in this study reported that clinical healthcare services for women with GBC remained accessible throughout the COVID-19 pandemic. While the availability of services was not deemed to be a concern for HCPs, the feelings of isolation, limited non-clinical support, and decreased access to services are recognised by HCPs to likely contribute to increased anxiety and uncertainty for women with GBC.

The findings offer valuable insights from HCPs, enhancing our understanding of patient experiences with the complex domain of GBC. They highlight key areas for future research to improve the quality of care for women diagnosed with GBC. Notably, research on the psychological aspects of these women, particularly mothers, is limited. HCPs identified a gap in understanding the psychological challenges these women face, emphasising the need for a comprehensive, women-centred approach with adequate psychological support.

Future research comparing women with GBC to other breast cancer patients could provide insights into their unique psychological distress. Understanding this could help HCPs better appreciate their experiences of motherhood and pregnancy. An in-depth examination of the psychological, emotional, and social needs of women with GBC and their families is essential for enhancing care and health outcomes.

### Implications for Clinical Practice

The findings show important implications for clinical practice concerning GBC. HCPs understand the trauma and unmet psychological needs of women diagnosed with GBC. There is a strong emphasis on providing routine psychosocial support, conducting comprehensive screenings, offering practical peer support groups, and establishing guidelines. Additionally, specialised training for nurses and multidisciplinary teams is crucial to effectively address both the clinical and psychological challenges these women encounter.

Moreover, clinical education emphasises the integration of psychological management and psychosocial care, particularly in relation to the interconnected issues of motherhood and cancer. It is essential to develop educational resources for women diagnosed with GBC, covering topics such as genetic considerations, fertility preservation, breastfeeding, surgical options, and treatment protocols. Targeted resources for nurses, the multidisciplinary team, and women with GBC can enhance communication and management of these complexities.

Other important considerations for clinical practice involve training nurses and the multidisciplinary team to recognise the time constraints and diverse treatment needs of women with GBC. This training is essential, as it underscores the vital role that HCPs play in advocating for these women throughout the decision-making process. Effective advocacy enhances their autonomy, satisfaction, and overall quality of care. Key areas for advocacy include scheduling treatments, timing surgeries, planning chemotherapy, and determining delivery dates, all of which are significantly influenced by the health of the baby and family dynamics.

## Conclusion

This study explored the narratives of 11 HCPs on their perceptions of the experiences of women diagnosed with GBC. The HCPs provided valuable insights into the significant psychological impact of GBC, highlighting the unique challenges that differentiate women with GBC from other breast cancer patients and their support needs. Additionally, the study revealed that the decision-making process for women with GBC involves essential elements, including information, support, and trust.

## Data Availability

The data used to support the findings of this study are available from the corresponding author upon request, and all data availability is subject to the research team’s discretion.
